# The comparison of socioeconomic status, perceived social support and mental status in women of reproductive age experiencing and not experiencing domestic violence in Iran

**DOI:** 10.5249/jivr.v10i1.983

**Published:** 2018-01

**Authors:** Roshanak Vameghi, Sedigheh Amir Ali Akbari, Hamid Alavi Majd, Firoozeh Sajedi, Homeira Sajjadi

**Affiliations:** ^*a*^Pediatric Neurorehabilitation Research Center, University of Social Welfare and Rehabilitation Sciences, Tehran, Iran.; ^*b*^Department of Midwifery, Faculty of Nursing and Midwifery, Shahid Beheshti University of Medical Sciences, Tehran, Iran.; ^*c*^Department of Biostatistics, Shahid Beheshti University of Medical Sciences, Tehran, Iran.; ^*d*^Social Determinants of Health Research Center, University of Social Welfare and Rehabilitation Sciences, Tehran, Iran.

**Keywords:** Domestic violence, Stress, Depression, Social support, Socioeconomic status

## Abstract

**Background:**

Given the significant health effects of domestic violence against women, the present study was conducted in 2016, in Tehran, Iran in order to compare the socioeconomic status, perceived social support and mental status in women of reproductive age experiencing and not experiencing domestic violence.

**Methods:**

This descriptive-analytical cross-sectional study was conducted on 500 women. The data collection tools used included questionnaires: demographic information, Socioeconomic, Beck’s Depression, Spielberger’s Anxiety, Cohen’s Perceived Stress, Sarason’s Perceived Social Support and WHO’s Domestic Violence Inventory.

**Results:**

The results showed that 43.2% of women said they had experienced at least one case of domestic violence, among which 16.4%, 15% and 36.6% of women had experienced physical, sexual and emotional-verbal types of violence, respectively. The mean age (p less than 0.001) and educational level (p=0/018) of violated women and their spouses (p less than 0.001) were lower than those of non-violated women. Furthermore, violated women experienced lower socioeconomic status (p less than 0.05), higher perceived stress (p less than 0.008), higher depression (p less than 0.001), and higher overt anxiety (0.002. They also perceived lower levels of social support (p less than 0.001).

**Conclusions:**

The issue of domestic violence was rather prevalent in the participants of the present study, particularly the younger, less educated and more socioeconomically deprived communities and families.

## Introduction

Violence has been an important issue in public health for about two decades and violence against women is considered one of the most serious social problems surpassing cultural, social and regional boundaries.^1,2^ The World Health Organization recognizes this problem as a global one observed in most countries and in people of all socioeconomic classes. The WHO has reported the prevalence of domestic violence to range from 15% in Japan to 71% in Ethiopia. Women of reproductive age appear to be the greatest victims of domestic violence.^3^ Given its significant contribution to physical injuries, mental illnesses and unfavorable outcomes in women, domestic violence is not only proposed as a major health problem for women, but is also considered a persisting hidden epidemic. About one third of the world’s population of women experience domestic violence.^4^

According to a WHO report, domestic violence is the most frequent violence committed against women and causes serious consequences. Domestic violence deaths often remain undetected due to the failure of the system to record the specific cause of death and also due to the number of deaths caused by suicide and drug abuse following domestic violence.^5^ Domestic violence indicates any violent behavior committed against someone by a family member that causes physical, psychological, social, economic and sexual harm and leads to injury, death, psychological trauma, hindered progress and deprivation.^6,7^

The results of other studies have shown that domestic violence is linked to a low economic status and a history of depression in women, and has an inverse relationship with the degree of education and employment status of both spouses, marital satisfaction and social support.^8,9^ The adverse effects and harms of domestic violence are greater in societies that impose social limitations on women and entail unfavorable socioeconomic conditions for them, as they do not provide women with the power to have control over these harms. In some developing countries such as Pakistan where there is a gender discrimination in areas such as education, health and employment, the level of domestic violence committed against women is higher.^8^

In developed countries, domestic violence is least common in families with a moderate to high socioeconomic status and higher levels of education and is most common for poorer women.^10^ Higher levels of education in both spouse shave been reported as a protective factor against violence. Higher levels of education in the husband appear to make him act more favorably toward his wife and commit less violence against her, probably due to his higher awareness of the social and familial duties of a man.^11^ Women’s level of education is also strongly related to violence.^12^ The results of some studies suggest that higher levels of education, better socioeconomic status and being married officially are factors that reduce domestic violence against women, while drinking, being younger, having less social support and premarital and extramarital relationships further contribute to domestic violence.^13^

From the perspective of health professionals violence against women is a major public health problem and the female victims of domestic violence often suffer from physical injuries and chronic conditions.^14^ Not only does violence threaten the individual’s health, but also it entails long-term unfavorable outcomes and leads to a low quality of life and lower use of healthcare services for these women, which continue even after the violence has ended.^10^ WHO considers violence against women as a main cause of anxiety, depression, suicidal thoughts and stress among women.^15^ Women experiencing domestic violence have significantly lower scores on emotional intelligence indicators.^16^ There is a positive impact of emotional intelligence on the life and psychological and social functioning.^17^

Tsirigotis and Łuczak reported that resilience of study women suffering domestic violence was lower than resilience of the general population not experiencing domestic violence. ^18^ Based on different studies, sexually-abused women experience higher degrees of anxiety and depression disorders. ^19^ Psychological complications, suicidal behaviors, drug abuse, posttraumatic stress disorder and obsessive-compulsive disorders have also been reported in these women, ^20,21,22^ as well as higher levels of stress.^23^

Given the importance domestic violence against women and its adverse health effects for women, families, children and the society^24,25^ and considering the lack of suffice entepidemiological studies on the its contributing factors in Iran and many other middle eastern countries, the present study was conducted to determine the status of domestic violence in Iranian women of reproductive age residing in Tehran and to compare some factors that the authors anticipated might have influence on or be influenced by the issue, based on literature, that is demographic factors, socioeconomic status, mental status and perceived social support,^9,20,23^ between women experiencing and not experiencing domestic violence.

## Methods 

The present descriptive-analytical cross-sectional study was conducted on 500 women visiting a number of medical centers affiliated to Shahid Beheshti University of Medical Sciences in Tehran in 2014. Once the project was approved by the Ethics Committee of the University of Social Welfare and Rehabilitation Sciences and the necessary permits were obtained, the women were selected through multistage random sampling. The municipal regions of Tehran covered by Shahid Beheshti University of Medical Sciences were first identified and the list of clinics in each region was then prepared, representing clusters. A number of clinics were selected randomly from each region in proportion to the regions’ population. The sample size from each clinic was then determined based on the population of visited women during the past year. Iranian women aged 18-35 who had no previous or current histories of known medical and mental diseases were included in the study. They reported no previous or current use of psychiatric medications and had not experienced any major adverse life events such as the death of first-degree relatives over the past year. 

The data collection tools used included a demographic information questionnaire, the Socioeconomic Inventory, Beck’s Depression Inventory, Spielberger’s Anxiety Inventory, Cohen’s Perceived Stress Scale, Zimet’s Perceived Social Support Inventory and the WHO Domestic Violence Inventory. 

The demographic information questionnaire was researcher-designed and ten faculty members confirmed its face and content validity. It included such demographic factors as the woman’s and her husband’s age, the woman’s and her husband’s level of education, the woman’s employment status and family income. The socioeconomic status of the subjects was assessed using the socioeconomic inventory designed by Garmaroudi et al. ^26^, with components including the subjects' level of education, the spouse’s level of education, ratio of home area to household size, price of the home per square meter, facilities and amenities (such as car and computer ownership) and family income. The correlation between these factors and the total score obtained in the inventory has been reported to be 0.87, and the test-retest reliability has been determined to be 0.96. The inventory provides a cut-off point of 16 for differentiating between favorable and unfavorable socioeconomic status. The maximum obtainable score is 48. Cohen’s scale for assessment of perceived stress is developed to measure perceived stress in the preceding month^27^ and is widely used in different countries and has been translated into different languages and standardized for use in different cultures. The present study used the 14-item version of the scale. The score obtained in this scale varies between 0 and 56 and higher scores indicate a higher degree of perceived stress. No cut-off points have been specified for this scale. Bastani et al. determined the reliability of the Persian version of the scale through measuring its internal consistency and calculated its Cronbach’s alpha as 0.74.^28^ Other studies using this scale in Iran have calculated its Cronbach’s alpha as 0.84-0.86.^29-32^ The present study calculated the reliability (internal consistency) of the scale as 0.88 and its test-retest reliability as 0.92. 

The Perceived Social Support Inventory was designed by Sara Son et al, (1983)^33^ and was then translated into Persian by Naseh et al, (2012). The validity and reliability of the inventory were measured and its internal consistency was confirmed with a Cronbach’s alpha of 0.95. ^34^ Other studies conducted in Iran have calculated the reliability of the inventory as 0.86-0.89.^35,36^ This inventory has also been used in studies conducted in other countries.^37,38^ The minimum and maximum scores that can be obtained in this inventory are 12 and 84. The present study calculated the reliability (internal consistency) of the scale as 0.89 and its test-retest reliability as 0.92. 

The State-Trait Anxiety Inventory (STAI) is an introspective psychological inventory consisting of 40 self-report items pertaining to anxiety affect. The anxiety scores obtained in this inventory range from a minimum of 20 to a maximum of 80, with scores of 20-40 indicating mild anxiety, 41-60 indicating moderate anxiety and 61-80 indicating severe anxiety. Numerous studies have determined the validity and reliability of inventory for measuring anxiety.^39-43^ The reliability of this inventory has been examined in two studies in Iran: one study conducted in Tehran calculated it as 0.911^44^ and the pther conducted in Mashhad as 0.95. ^45^ The present study calculated the test-retest reliability of the inventory as 0.94. 

Beck’s Depression Inventory has 21 items with a score ranging from 0 to 63. Different studies have confirmed the reliability of this inventory. It has also been standardized for use in Iran where its cut-off point has been determined to be 9 and the internal consistency of the inventory was confirmed for use in Iran with a Cronbach’s alpha of 0.87 and its reliability was then calculated to be 0.74.^46-48^ The present study calculated the test-retest reliability of the inventory as 0.92.

The operational definition of domestic violence in this study was any violence committee against a woman by her husband. The Domestic Violence Inventory developed by the WHO was used in this study to assess the physical, sexual and emotional dimensions of violence. Any woman who responds ‘yes’ to at least one of the items on physical, sexual and emotional violence is considered violated. Researchers have assessed the reliability of the inventory in Iran and have calculated its Cronbach’s alpha as 0.92, 0.89 and 0.88 for the physical, emotional and sexual dimensions of violence.^49^ The test-retest reliability of the inventory was calculated to be 0.82 in the present study.

The above-mentioned tools were distributed among the participants after they were briefed on the objectives of the study and once they offered their consent for participation. To comply with the ethics of research, participants who showed a high level of anxiety, stress or depression or who needed abuse counseling were referred to relevant health authorities at the end of the study for receiving therapeutic and supportive services. 

The data obtained were analyzed in SPSS-19 using Mann-Whitney’s U test, and the independent t test. The level of statistical significance was determined as 0.05.

## Results

The results showed that 43.2% of women said that they had experienced at least one case of domestic violence. The highest frequency was related to emotional-verbal abuses (Table 1). Some women reported that they had experienced two or three types of violence together. 

**Table 1:** Frequency of domestic violence in women referring to health-care centers affiliated to Shahid Beheshti University of Medical Sciences in 2014 in terms of different types and totally.

Type of domestic Violence	Frequency	percent
**Physical**	82	16.4
**Sexual**	75	15
**Emotional-Verbal**	183	36.6
**Total**	216	43.2

There was a significant difference between the violated and non-violated groups in terms of the mean age of women and the mean age of their husbands, such that violence occurred more in women of younger age groups, as well as in younger husbands (p <0.001). There was also a significant difference between the violated and non-violated groups in terms of the educational level of women and their spouses. Women with lower educational level (high school level and lower) said that they had experienced violence more often than other women. Significant difference was also seen in frequency of violence exerted by husbands with different educational levels, those with high school level and lower exhibiting the highest frequency (p <0.05) (Table 2).

**Table 2:** Comparison of some demographic factors in women referring to health-care centers affiliated to Shahid Beheshti University of Medical Sciences in 2014, experiencing and not experiencing domestic violence.

Variables	Groups	With domestic violence N=216	Without domestic violence N=284		Results
**Women’s age (mean±SD)**		27.41±4.32	28.83±3.87		**P<0.001**
					**t=3.846**
**Husband’s age (mean±SD)**		32.17±5.15	34.49±5.59		**P<0.001**
					**t=4.776**
**Women’s education**	**Level**	**Frequency(percent)**	**Frequency(percent)**	**Total**	**P<0.01**
	Primary	13 (6.1)	36 (12.7)	49 (9.8)	
	High school	155 (71.7)	136 (47.8)	291 (58.2)	
	Diploma	40 (18.5)	95 (33.5)	135 (27)	
	College	8 (3.7)	17 (6)	25 (5)	
	Total	216 (100)	284 (100)	500 (100)	
	Mean Rank	235/06	262/24		
**Husband’s education**	Primary	21(9.7)	27(9.5)	48(9.6)	**P<0.001**
	High school	150(69.4)	152(53.5)	302(60.4)	
	Diploma	38(17.7)	78(27.5)	116(23.2)	
	College	7(3.2)	27(9.5)	34(6.8)	
	Total	216(100)	284(100)	500(100)	
	Mean Rank	228.46	267.26		

There were significant differences in frequency of experiencing violence between employed and unemployed women. Our results showed that women living in families with lower income and having lower socioeconomic status were violated more frequently than others (p<0.05) (Table 3).

**Table 3:** Comparison of socioeconomic status of women referring to health-care centers affiliated to Shahid Beheshti University of Medical Sciences in 2014, experiencing and not experiencing domestic violence.

Variables	Groups	With domestic violence N=216	Without domestic violence N=284		Results
**Mean Family income per month Million Rials-Iranian currency) ± SD**		10025462.96 ± 3151308.295	10607394.36 ± 3380976.959		**P=0.04**
**Women’s Emplyment (frequency-percent)**	Not Employed (Housewife)	209 (44.6)	260 (55.4)	469 (100%)	**P=0.01**
	Employed	7 (22.6)	24 (77.4)	31(100%)	**RR=2.756 CI(1.165-6.522)**
**Socio-economic status (mean±SD)**		20.75 ± 6.49	22.49 ± 7.54		**P<0.05**
					**RR=1.135 (0.39-0.912)**

In terms of the correlation between women’s stress and anxiety with their report of domestic violence, the results showed that the mean score of perceived stress was significantly higher in abused women (p=0.005). In regard to anxiety, most women in both abused and non-abused groups showed moderate levels of anxiety, although abused women showed higher trait anxiety (Table 4).

**Table 4:** Comparison of stress score and anxiety status in women referring to health-care centers affiliated to Shahid Beheshti University of Medical Sciences in 2014, experiencing and not experiencing domestic violence.

Variables	Groups	With domestic vio-lence N=216	Without domestic violence N=284	Total Frequency (percent)	Results
**Trait Anxiety (frequency and percent)**	**Mild**	42 (19.4)	89 (31.3)	131 (26.2)	**P=0.002 Mann-Whitney u**
	**Moderate**	171 (79.2)	194 (68.3)	365 (73)	
	**Severe**	3 (1.4)	1 (0.4)	4 (0.8)	
	**Total**	216 (100)	284 (100)	500 (100)	
	**Mean Rank**	268.34	236.93		
**State (frequency and percent) Anxiety**	**Mild**	29 (13.4)	30 (10.6)	59 (11.8)	**NS Mann-Whitney u**
	**Moderate**	176 (81.5)	249 (87.7)	425 (85)	
	**Severe**	11 (5.1)	5 (1.8)	16 (3.2)	
	**Total**	216 (100)	284 (100)	500 (100)	
**Stress Score (mean ± SD)**	25.59 ± 8.24		23.58 ± 8.46		**P=0.008 Inde-pendent t-test**

As can be seen in Table 5, this study also revealed that women experiencing violence suffered significantly higher levels of depression, in all three levels of severity.

**Table 5:** Comparison of depression status in women referring to health-care centers affiliated to Shahid Beheshti University of Medical Sciences in 2014, experiencing and not experiencing domestic violence.

Variables	Groups	With domestic violence N=216	Without domestic violence N=284	Total Frequency (percent)	Results
**Depression (frequency and percent)**	**None**	78 (36.1)	177 (62.3)	255 (51)	**P=0.001**
	**Mild**	72 (33.3)	71 (25)	143 (28.6)	
	**Moderate**	47 (21.8)	25 (8.8)	72 (14.4)	
	**Severe**	18 (8.3)	9 (3.2)	27 (5.4)	
	**Extremely Severe**	1 (0.5)	2 (0.7)	3 (0.6)	**RR=2.92**
	**Total**	216 (100)	284 (100)	500 (100)	**CI (2.02-4.22)**
	**Mean Rank**	292.41	218.93		

In terms of the scores obtained for social support, the highest score in both groups was related to the support received from family members. However, there was a significant difference between the two groups in terms of the social support received from the husband, friends, and family members, separately, as well as totally such that the non-violated women enjoyed significantly higher social support than violated women from all three sources (Table 6). In terms of different degrees of total score of social support, the non-violated group had reported significantly higher levels of moderate and high social support, whereas the violated group reported significantly higher levels of low social support (Table 7).

**Table 6:** Comparison of social support received from husband, friends and family in women referring to health-care centers affiliated to Shahid Beheshti University of Medical Sciences in 2014, experiencing and not experiencing domestic violence.

Variables	Groups	With domestic violence N=216	Without domestic violence N=284	Results
**Domain of Social Support (score)**	**Husband**	20.79 ± 5.90	23.72 ± 4.49	P=0.001
	**friends**	21.35 ± 5.94	23.70 ± 4.65	P=0.001
	**family**	15.58 ± 7.39	18.66 ± 7.55	P=0.001
**Total Social support**		57.72 ± 15.29	66.08 ± 13.39	P=0.001

**Table 7:** Comparison of different levels of social support in women referring to health-care centers affiliated to Shahid Beheshti University of Medical Sciences in 2014, experiencing and not experiencing domestic violence.

Variables	Groups	With domestic violence N=216	Without domestic violence N=284	Total Frequency(percent)	Results
**Total Social Support (frequency and percent)**	**Low**	56 (25.9)	30 (10.6)	86 (17.2)	**P=0.001**
	**Moderate**	96 (44.5)	113 (39.8)	209 (41.8)	
	**High**	64 (29.6)	141 (49.6)	205 (41)	
	**Total**	216 (100)	284 (100)	500 (100)	
	**Mean Rank**	214.09	278.19		

## Discussion

The results obtained showed that 43.2% of the participants had reported domestic violence. The prevalence of domestic violence in any society shows that the physical, emotional and mental health of women is threatened in that society. It also indicates that the physical, emotional and mental health of the victims, whoever they may be, that is women or men, is threatened in that society. The WHO has reported domestic violence against women to range from 14% to 71% in different countries and considers it the most common form of violence committed against women.^3^ Al-Atrushi et al. reported the frequency of domestic violence against women as 58.6% in 2013 in Baghdad. ^50^ The frequency of domestic violence committed against women was reported to be 58% in 2006 in Bangladesh,^51^ 52% in 2006 in Turkey,^52^ 14.3% in 2007 in Japan ^53^ and 97.5% in 2007 in Pakistan. ^54^ In Ethiopia in 2013, 7 even out of every 10 women (70 %) was reported to have experienced domestic violence.^55^ These rather high figures signify the need to address this critical and prevalent issue by national authorities. Moreover, the distinctive design of the studies and the data collection tools used for measuring violence may have affected the results. 

The results of the present study showed that the mean age of both the violated women and their abusive husbands was lower compared to the couples in the non-violated group. These results are consistent with the results obtained by Shakerinejad et al. in 2013 in Zanjan city in Iran.^56^ The reason for this higher frequency among younger couples may be their immaturity and lack of experience for conducting a balanced and successful family life, which may end in violence. Younger couples may also be a cause for the impulsive and aggressive behaviors committed by one of them against the other in confronting with life’s difficulties and challenges. Another possible reason for a higher incidence of violence in the younger generation of couples may be the confrontation of young couples in developing countries such as Iran, with the paradox of traditional cultural values which supports the idea of male dominance and excellence, versus the newly emerging modern culture of struggle for sexual equalization and against sexual discrimination, that when not resolved at the social level, ultimately leads to disagreement and perhaps violence within the family. Yet another possibility is that younger women, who tend to have younger spouses, are more sensitive to violence exerted from their husbands and act more openly and report more overtly about it than older women, who may even consider such violence as normal and unnecessary to report.

There were no significant differences between the two groups of violated and non-violated women examined in this study in terms of employment and unemployment. Although WHO has also reported similar findings,^13^ the reason for this insignificance in the present study may also be that most of the recruited women were housewives.

The present study revealed a lower socioeconomic status in abused than in non-abused women, which is consistent with the results of a study conducted by Abramsky et al. in 2011.^13^ Djikanovic et al. in 2010 too conducted a study in Serbia and showed that domestic violence is linked to socioeconomic status and family income.^57^ Also, Babu et al. in 2007 conducted a study in India and reported a lower level of domestic violence in women living in high-income families.^58^ Tolman et al. in 2001 however, found no relationships between socioeconomic status and domestic violence in women from Michigan, which is inconsistent with the results obtained in the present study. ^59^ Perhaps, the reason for this positive correlation is that violence may be a response to economic problems, as low-income families usually experience tensions and pressures exerted on them by unemployment and poverty^60^ which may ultimately express itself in the form of aggression and violence.

The present study found that the level of education was significantly lower in the violated than in the non-violated women, which is consistent with the results of some other studies conducted in Serbia in 2010 and in Vietnam in 2008.^57,61^ In terms of the spouse’s level of education, consistent with our findings Ali et al.in 2014 in Eastern Sudan showed a correlation between the husband’s level of education and domestic violence committed against women, such that men with lower levels of education committed more violence against their wives than other men.^62^ It may be that women with higher levels of education, who most probably marry men with higher levels of education, have greater potential and opportunity for independence and decision-making power, especially with respect to family issues, and therefore experience less instances of domestic violence.

The present study found violated women to experience significantly higher levels of trait anxiety and stress, which is consistent with the results obtained by Ali et al. in 2014.^62^ Jaquier et al. in 2014 also revealed that 60% of abused women experience post-traumatic stress disorders. ^63^ Renner et al. in 2009 too found a relationship between domestic violence and stress in women.^64^ Vinck et al. in 2013 examined women who were physically abused by their husbands in Libya and found that 12.6% suffer from PTSD and 10.2% from depression.^65^ Another study conducted on 117 women suffering from sexual and physical domestic violence in Uganda showed a correlation between PTSD and sexual abuse.^66^ In a study conducted in Jordan, violated women showed significantly higher levels of stress, anxiety and depression compared to the women in the control group.^67^

The present study found significantly higher levels of depression among women who had experienced domestic violence, than those who had not. Lagdon et al, in 2014 showed that women abused by their partners are more prone to mental problems such as stress and depression.^68^ Kader Maideen. et al, in 2014 found that the abused women experience depression 7.09 times more than the non-abused.^69^ Moreover, a study conducted by Teng et al, in 2014 showed that the abused tend to experience higher levels of depression.^70^ Other studies too have confirmed the relationship between violence and depression, which is consistent with the results of the present study.^71-73^

The abused women examined in the present study perceived lower social support compared to the non-abused women. A study conducted by Teng et al, in 2014 showed that abused women tend to perceive lower levels of social support. ^70^ Khosla et al. showed that women who had no social support or families to support them were more exposed to domestic violence.^74^ The results obtained by Gillum et al. in 2006 are also consistent with the results of the present study with respect to social support and domestic violence. ^9^ The results of several other studies also indicate that social support acts as an external source that reduces domestic violence in families.^13,56,74^ This correlation reflect show supportive relationships outside the family can encourage healthy behaviors inside the family, and how presence of social support may act as a preventive and an immunizing factor for the commitment of violence towards women from their spouses.

## Conclusion

Overall, the results of the present study demonstrated an impressive figure for the prevalence of domestic violence against Iranian women, which was more often verbal-emotional in type and which was shown to be especially prevalent in younger couples with lower educational and lower socioeconomic status. This phenomenon tended to occur less frequently whenever higher social support existed and was perceived by the woman. Like many other women in other parts of the world, violated Iranian women showed higher levels of depression, stress, and severe degrees of anxiety. Given the significant role of women in the establishment of balance, emotional attachment and psychosocial health for the child, the family and the society, which can only be accomplished when they live in a peaceful and safe environment free of violence, and when their physical, mental and emotional health is ensured, it is the responsibility of national and international health authorities to address the critical issue of domestic violence in Iran and most probably in many similar countries of the region, and to devise plans for its prevention and control, particularly with regard to younger, less educated and more socioeconomically deprived communities and families. 

**Acknowledgements:**

The authors sincerely thank all of the health centers and women for their assistance.
